# Accuracy of Anthropometric Equations for Estimating Body Fat in Professional Male Soccer Players Compared with DXA

**DOI:** 10.1155/2018/6843792

**Published:** 2018-03-14

**Authors:** Juan R. López-Taylor, Roberto G. González-Mendoza, Alejandro Gaytán-González, Juan Antonio Jiménez-Alvarado, Marisol Villegas-Balcázar, Edtna E. Jáuregui-Ulloa, Francisco Torres-Naranjo

**Affiliations:** ^1^Institute of Applied Sciences for Physical Activity and Sport, Department of Human Movement Sciences, Education, Sport, Recreation and Dance, University Health Sciences Center, University of Guadalajara, Av. Revolución 1500, 44430 Guadalajara, JAL, Mexico; ^2^Department of Human Reproduction, Infantile Growth and Development, University Health Sciences Center, University of Guadalajara, Hospital 320, 44280 Guadalajara, JAL, Mexico; ^3^State Secretary of Health Jalisco, Dr Baeza Alzaga 107, 44100 Guadalajara, JAL, Mexico; ^4^Center of Body Composition and Bone Research, Av. Mexico 2481, 44650 Guadalajara, JAL, Mexico

## Abstract

**Background:**

There are several published anthropometric equations to estimate body fat percentage (BF%), and this may prompt uncertainty about their application.

**Purpose:**

To analyze the accuracy of several anthropometric equations (developed in athletic [AT] and nonathletic [NAT] populations) that estimate BF% comparing them with DXA.

**Methods:**

We evaluated 131 professional male soccer players (body mass: 73.2 ± 8.0 kg; height: 177.5 ± 5.8 cm; DXA BF% [median, 25th–75th percentile]: 14.0, 11.9–16.4%) aged 18 to 37 years. All subjects were evaluated with anthropometric measurements and a whole body DXA scan. BF% was estimated through 14 AT and 17 NAT anthropometric equations and compared with the measured DXA BF%. Mean differences and 95% limits of agreement were calculated for those anthropometric equations without significant differences with DXA.

**Results:**

Five AT and seven NAT anthropometric equations did not differ significantly with DXA. From these, Oliver's and Civar's (AT) and Ball's and Wilmore's (NAT) equations showed the highest agreement with DXA. Their 95% limits of agreement ranged from −3.9 to 2.3%, −4.8 to 1.8%, −3.4 to 3.1%, and −3.9 to 3.0%, respectively.

**Conclusion:**

Oliver's, Ball's, Civar's, and Wilmore's equations were the best to estimate BF% accurately compared with DXA in professional male soccer players.

## 1. Introduction

Body fat percentage (BF%) is probably the most evaluated body composition component in sports [1]. In soccer, BF% estimation is part of the habitual assessment [2], because it is related to exercise performance [3, 4], may be useful in seasonal monitoring [5, 6], and may help compare players in different playing positions [3, 7–9] and experience [10, 11].

Several methods are available when evaluating BF% [1, 12]. Dual-energy X-ray Absorptiometry (DXA) is a laboratory method used to assess body composition, which is increasingly being used to evaluate BF% in soccer players [6, 8–10] due to its practicality and accuracy compared with other reference methods [1, 13, 14].

Conversely, anthropometry, one of the most popular field methods in assessing body composition, is used to estimate BF%, typically with regression equations obtained from other laboratory methods [1, 12]. These equations have been developed in athletic and nonathletic populations and they have a too wide variability of estimation when applied to the same subject/population. Similarly, several studies have investigated the accuracy of these equations in estimating BF% in specific populations [15–21]. However, little is known about their accuracy when they are applied on professional male soccer players [16, 20].

Therefore, the purpose of this study was to analyze the accuracy of several anthropometric equations that estimate BF% utilizing DXA as the reference method in professional male soccer players. Since there is a great amount of anthropometric equations [1], we decided to analyze some of these separately as follows:

- Anthropometric equations developed in athletic populations (AT)
- Anthropometric equations developed in nonathletic populations (NAT).

## 2. Methods

### 2.1. Study Design

This was a cross-sectional observational study. Participants were evaluated between the years 2009 and 2015 as part of their medical screening.

### 2.2. Participants

The sample consisted of 131 professional male soccer players (121 Mexicans, 9 South Americans, and 1 Spaniard) from the 2nd Professional Mexican Soccer League. Their age, body mass, and height ranged from 18 to 37 years, 53.3 to 93.6 kg, and 165.3 to 190.8 cm, respectively.

### 2.3. Procedures

Participants attended our laboratory at 9:00 am, after a two-hour fasting. They were instructed to avoid any exercise prior to their evaluations. Each participant was evaluated by a whole body DXA scan and a complete anthropometric assessment, both done on the same day. Body fat percentage was estimated for each subject through 14 AT (Table 1) and 17 NAT anthropometric equations (Table 2) and compared with the body fat percentage obtained with DXA.

#### 2.3.1. Anthropometric Measurements

These measurements consisted of body mass to nearest 0.1 kg (TBF-410, Tanita, Tokyo, Japan), height to nearest 0.1 cm (Seca 213, Seca, Hamburg, Germany), 10 skinfolds (triceps, subscapular, biceps, chest, axilla, iliac crest, supraspinal, abdomen, thigh, and calf) to nearest 0.1 mm (Harpenden, Baty International, United Kingdom), waist circumference with a metallic tape (to nearest 0.1 cm), and elbow diameter (Campbell 10, Rosscraft, Canada) to nearest 0.05 cm. All measurements were evaluated by trained personnel following standardized procedures. All measurements were evaluated following the ISAK protocol [22, 23], but chest and axilla skinfolds were evaluated following Lohman's proposed sites [24]. The intraobserver technical error of measurement in our laboratory is ≤5% for skinfolds and ≤1% for all other measurements. The interobserver technical error of measurement in our laboratory is ≤7.5% for skinfolds and ≤1.5% for all other measurements.

#### 2.3.2. DXA Scanning

A whole body scan was performed for each subject with a DXA fan beam equipment (Hologic QDR4500 Explorer, Massachusetts, USA). The equipment was calibrated daily following the manufacturers indications. All scans were analyzed with the software for Windows® Hologic QDR v 12.1 (1986–2002©, Hologic Inc.). The zone of head was excluded from the scan in order to calculate the body fat percentage. The difference between DXA whole body mass and body mass on scale was on average −1.0 ± 0.7 kg. The whole procedure was executed by a certified DXA technician. His technical error of measurement was 0.4 ± 2.9% for body fat, −0.2 ± 1.2% for lean body mass, and 0.0% ± 0.5% for total mass.

#### 2.3.3. Anthropometric Equations Developed in Athletic Populations

Body fat percentage was estimated with 14 AT anthropometric equations for each participant. These equations estimate body density [25–30], body fat percentage [16, 18, 31, 32], or body fat mass [33] (Table 1).

#### 2.3.4. Anthropometric Equations Developed in Nonathletic Populations

Body fat percentage was estimated with 17 NAT anthropometric equations for each participant. These equations estimate body density [34–43], body fat percentage [44–48], and body fat mass [49] (Table 2).

Siri's equation [50] was utilized to transform the anthropometrical estimation of the body density to BF%. If the equations estimated body fat mass, the BF% was calculated using scale body mass.

#### 2.3.5. Statistical Analysis

In order to determine which equations estimated the BF% different than the one obtained with DXA, all anthropometrical BF% estimations were tested with the Kruskal-Wallis and Dunn's post hoc test, considering a significance level of *p* < 0.05. Taking only the equations that showed no significant difference with DXA, we carried out a modified Bland-Altman analysis. Mean difference, linear correlation, and 95% limits of agreement for each equation were calculated [51]. Variables with normal distribution (D'Agostino-Pearson test) are expressed as mean ± SD and as median (25th–75th percentile) if they were not normally distributed. All analyses were carried out with GraphPad® Prism 7 for Windows (GraphPad Software, California, USA). Homoscedasticity was confirmed (*p* > 0.05) with Breusch-Pagan test after eliminating one outlier from the analysis for all Bland-Altman comparisons (SPSS® v24, IBM Corp, Armonk, NY, USA).

## 3. Results

### 3.1. Participants

The age of participants was 23.2 (20.5–26.8 y), body mass 73.2 ± 8.0 kg, height 177.5 ± 5.8 cm, BMI 23.2 ± 1.9 kg/m^2^, and DXA BF% 14.0 (11.9–16.4). The skinfold thicknesses are presented in Table 3.

### 3.2. Body Fat Percentage Estimation with AT Anthropometric Equations

From the 14 anthropometric equations analyzed, five showed no significant differences compared with DXA (Table 4). From these equations, those with the narrowest limits of agreement with DXA were the proposed by Oliver [32] and Civar [18], followed by Zuti [30], Forsyth (4SKF), and Forsyth (2SKF) [25] equations. These last three equations showed small mean differences with DXA but wider limits of agreement (Table 5). Oliver's equation showed slightly narrower limits of agreement and a smaller mean difference compared with Civar's equation (Table 5).

### 3.3. Body Fat Percentage Estimation with NAT Anthropometric Equations

From the 17 anthropometric equations analyzed, seven showed no significant differences compared with DXA (Table 6). From these equations, those with the narrowest limits of agreement and smallest mean differences with DXA were Ball's [44] and Wilmore's [43] equations, followed by Durnin-R [34], van der Ploeg [48], Durnin-W [35], Lean [38], and Garcia [49] equations. These last five equations showed wider limits of agreement and/or higher mean differences with DXA (Table 7). Ball's equation showed slightly smaller mean difference and narrower limits of agreement with DXA than Wilmore's equation (Table 7).

## 4. Discussion

The purpose of this study was to analyze the accuracy of BF% obtained through several anthropometric equations and that measured with DXA in professional male soccer players. We only found two other studies that addressed the same issue in soccer players [16, 20]. In the study of Reilly et al. [16] they evaluated professional male soccer players from the English premier league and settled DXA as the reference method. They reported that Withers et al. [29] and Durnin and Womersley (Durnin-W) [35] equations showed the narrowest limits of agreement with DXA in their sample. However, this differs with our results, where Withers' equation was significantly different to DXA (Table 4). On the other hand, Durnin-W equation was similar to DXA but was not the one with the highest agreement (Table 7). The differences observed between Reilly et al. study and our results may be explained by the samples competitive level (first division versus second division). To our knowledge there is no other study comparing BF% obtained with anthropometric equations employing DXA as the reference method.

Da Fonseca et al. [20] studied professional male soccer player from Brazil; however, they compared the body density obtained through anthropometric equations with that evaluated with hydrostatic weighting. They reported that Jackson's equations (using three and seven skinfolds) [36] and Lohman's equation [39] did not differ significantly with the reference method, and Lohman's equation showed the lowest estimated standard error. In the present study, we found these three equations differed significantly with DXA (Table 6).

Other studies have compared anthropometric equations with hydrostatic weighting in different athletic populations [17, 18, 21]. The study of Sinning et al. [17] reported that Jackson's equation (seven skinfolds) [36] was not different to the reference method and showed a low SEE in collegiate male athletes. Civar et al. [18] reported that the Lohman [39] and Durnin and Womersley (Durnin-W) [35] equations did not differ with the reference method, but Durnin-W equation showed a higher correlation and a lower SEE than Lohman's equation in college males that practiced different sports. Smith and Mansfield [21] evaluated male football players and reported that Jackson's equation (three skinfolds) [36] did not differ significantly with the reference method. Finally, Knechtle et al. [15] employed a different reference method; they studied to ultra-endurance male athletes and evaluated their BF% with bioelectrical impedance. They reported that Ball's equation [44] showed the highest agreement with their reference method.

Different results between previous studies and ours may be explained by the different population in which they were validated (football players, college age men, etc.) and the reference method used.

From the five AT anthropometric equations that did not differ significantly with DXA, four were validated with hydrostatic weighting [18, 25, 30] and one was validated with DXA [32]. This last one is with highest agreement with DXA in our study (Tables 5 and 7). On the other hand, from the seven NAT anthropometric equations that did not differ significantly with DXA, five were developed with densitometric methods [34, 35, 38, 43, 48] and two with DXA [44, 49]. Even though these equations were developed with a different body composition method, they were able to estimate BF% with a reasonable degree of agreement with DXA in our sample (Table 7).

Oliver's equation [32] showed the narrowest limits of agreement with DXA in our population (Tables 5 and 7). Oliver's equation was developed in a large sample of college football players. This equation employs the sum of seven skinfolds, including limbs and trunk measurements. The accuracy showed with this equation in our study could be explained by the number of skinfold thicknesses employed and the whole body representativeness from these skinfolds and because they also employed DXA as the reference method.

Wilmore's equation was developed in male university students (aged 16 to 37 years) [43]. Surprisingly, Although it employs only two skinfolds (abdomen and thigh), it had a higher agreement than those that used more measurements.

In a practical way, Wilmore's equation may be more useful because it accurately estimated BF% using few variables compared to the other equations. From another perspective, we recommend Oliver's equation, as it had higher agreement with DXA, and because it employs seven skinfolds it may offer a better whole body perspective.

Some of the limitations of this study were as follows:

1. The results of our reference method (DXA) may differ from other reference methods; however several studies have reported that DXA is a practical and accurate method for assessing BF% [1, 12–14].
2. The protocols from the original studies may differ with the one we followed to evaluate the anthropometric measurements; however we tried to keep the anatomical sites as similar as possible.
3. The instruments we used to evaluate the anthropometric measurements also differed from some of the authors' original studies.

Some of our strengths were as follows:

1. All our personnel (for anthropometric assessments and DXA scanning) are certified to do this kind of evaluations.
2. This study has a large sample.
3. We included many AT and NAT anthropometric equations.

## 5. Conclusions

In this study, we found that Oliver's (AT), Ball's (NAT), Civar's (AT), and Wilmore's (NAT) equations had the highest agreement with DXA for estimating BF% in professional male soccer players. These equations can be used alternatively to DXA for estimating BF% in a cross-sectional way. It remains to be seen if these equations are useful for monitoring changes in BF% over time. These issues deserve further research.

## Figures and Tables

**Table 1:** Analyzed anthropometric equations (developed in athletic populations).

Author	Equation^a^
Forsyth 2SKF [25]	BD = 1.103 − (0.00168 *∗* Sb) − (0.00127 *∗* Ab)
Forsyth 4SKF [25]	BD = 1.10647 − (0.00162 *∗* Sb) − (0.00144 *∗* Ab) − (0.00077 *∗* T) + (0.00071 *∗* Ax)
Pascale [26]^b^	BD = 1.088468 − (0.007123 *∗* Ax) − (0.004834 *∗* Ch) − (0.005513 *∗* T)
Thorland 3SKF [27]	BD = 1.1136 − (0.00154 *∗*Σ[T, SB, Ax]) + (0.00000516 *∗*Σ[T, SB, Ax]^2^)
Thorland 7SKF [27]	BD = 1.1091 − (0.00052 *∗*Σ[T, Sb, Ax, IC, Ab, Th, Ca]) + (0.00000032 *∗*Σ[T, Sb, Ax, IC, Ab, Th, Ca]^2^)
White [28]	BD = 1.0958 − (0.00088 *∗* IC) − (0.0006 *∗* Th)
Withers [29]	BD = 1.0988 − (0.0004 *∗*Σ[T, Sb, B, Sp, Ab, Th, Ca])
Zuti [30]	BD = 1.0806 − (0.001187 *∗* WC) − (0.001076 *∗* Ch) + (0.015306 *∗* WD)
Evans 3SKF [31]	BF% = 8.997 + (0.24658 *∗*Σ[Ab, Th, T]) − (6.343 *∗* Sex) − (1.998 *∗* Race)
Evans 7SKF [31]	BF% = 10.566 + (0.12077 *∗*Σ[Sb, T, Ch, Ax, IC, Ab, Th]) − (8.057 *∗* Sex) − (2.545 *∗* Race)
Oliver [32]	BF% = 3.53 + (0.132*∗*Σ[Ch, T, Sb, Ax, IC, Ab, Th])
Reilly [16]	BF% = 5.174 + (0.124 *∗* Th) + (0.147 *∗* Ab) + (0.196 *∗* T) + (0.13 *∗* Ca)
Civar [18]	BF% = (0.432 *∗* T) + (0.193 *∗* Ab) + (0.364 *∗* B) + (0.077 *∗* BM) − 0.891
Stewart [33]	BFM = (331.5 *∗* Ab) + (356.2 Th) + (111.9 *∗* BM) − 9108

BF%: body fat percentage; Ab: abdomen skinfold; Ax: axilla skinfold; B: biceps skinfold; BD: body density; BFM: body fat mass (g); BM: body mass (kg); Ca: calf skinfold; Ch: chest skinfold; IC: iliac crest skinfold; Sb: subscapular skinfold; SKF: skinfolds; Sp: supraspinal skinfold; T: triceps skinfold; Th: thigh skinfold; WC: waist circumference (cm); WD: wrist diameter (cm). ^a^All skinfolds are in millimeters, unless otherwise stated. ^b^Skinfolds in centimeters. Age: years. Sex: male 1 and female 0. Race: black 1 and white 0.

**Table 2:** Analyzed anthropometric equations (developed in nonathletic populations).

Author	Equation^a^
Durnin-R [34]	BD = 1.161 − (0.0632 *∗* log [ΣB, T, Sb, IC])
Durnin-W [35]	18-19 y → BD = 1.162 − (0.063 *∗* log [ΣB, T, Sb, IC])
	20–29 y → BD = 1.1631 − (0.0632 *∗* log [ΣB, T, Sb, IC])
	30–39 y → BD = 1.1422 − (0.0544 *∗* log [ΣB, T, Sb, IC])
Jackson 3SKF [36]	BD = 1.10938 − (0.0008267 *∗*Σ[Ch, Ab, Th]) + (0.0000016 *∗*Σ[Ch, Ab, Th]^2^) − (0.0002574 *∗* Age)
Jackson 7SKF [36]	BD = 1.112 − (0.00043499 *∗*Σ[Ch, Ax, T, Sb, Ab, IC, Th]) + (0.00000055 *∗*Σ[Ch, Ax, T, Sb, Ab, IC, Th]^2^) − (0.00028826 *∗* Age)
Katch [37]	BD = 1.09665 − (0.00103 *∗* T) − (0.00056 *∗* Sb) − (0.00054 *∗* Ab)
Lean [38]	BD = 1.1862 − (0.0684 *∗* log [ΣB, T, Sb, IC]) − (0.000601 *∗* Age)
Lohman [39]	BD = 1.0982 − (0.000815 *∗*Σ[T, Sb, Ab]) + (0.0000084 *∗*Σ[T, Sb, Ab]^2^)
Nagamine [40]	BD = 1.0913 − (0.00116 *∗*Σ[T, Sb])
Pollock [41]	BD = 1.09716 − (0.00065 *∗* Ch) − (0.00055 *∗* Sb) − (0.0008 *∗* Th)
Sloan [42]	BD = 1.1043 − (0.001327 *∗* Th) − (0.00131 *∗* Sb)
Wilmore [43]	BD = 1.08543 − (0.000886 *∗* Ab) − (0.0004 *∗* Th)
Ball [44]	BF% = 0.465 + (0.18 *∗*Σ[Ch, Ax, T, Sb, Ab, IC, Th]) − (0.0002406 *∗*Σ[Ch, Ax, T, Sb, Ab, IC, Th]^2^)+ (0.06619 *∗* Age)
Eston [45]	BF% = (0.12 *∗*Σ[B, T, Sb, IC]) + (0.36 *∗*Σ[Ca, Th]) + 1.61
Leahy [46]	BF% = (Age *∗* 0.1) + (7.6 *∗* log T) + (8.8 *∗* log Ax) + (11.9 *∗* log Sp) − 11.3
Peterson [47]	BF% = 20.94878 + (0.1166 *∗* Age) − (0.11666 *∗* Height) + (0.42696 *∗*Σ[T, Sb, IC, Th]) − (0.00159 *∗*Σ[T, Sb, IC, Th]^ 2^)
Van der Ploeg [48]	BF% = (0.183 *∗*Σ[T, Sb, B, IC, Sp, Ab, Th, Ca, Ax]) + (0.098 *∗* Age) − (0.00021 *∗*Σ[T, Sb, B, IC, Sp, Ab, Th, Ca, Ax]^2^) − 0.85
Garcia [49]	BFM = (WC *∗* 0.397) + (6.568 *∗* [log T + log Sb + log Ab]) − 40.75

BF%: body fat percentage; Ab: abdomen skinfold; Ax: axilla skinfold; B: biceps skinfold; BD: body density; BFM: body fat mass (kg); Ca: calf skinfold; Ch: chest skinfold; IC: iliac crest skinfold; Sb: subscapular skinfold; SKF: skinfolds; Sp: supraspinal skinfold; T: triceps skinfold; Th; thigh skinfold; WC: waist circumference (cm); y: years. ^a^All skinfolds are in millimeters, unless otherwise stated. Age: years. Height: centimeters.

**Table 3:** Subjects' skinfold thicknesses (*n* = 131), mm.

	Median (25th–75th percentile)
Triceps	7.5 (6.0–9.6)
Subscapular	9.6 (7.8–11.5)
Biceps	3.8 (3.1–4.5)
Chest	7.2 (5.7–9.4)
Axilla	8.0 (6.1–10.3)
Iliac Crest	13.9 (9.9–20.0)
Supraspinal	7.8 (6.3–11.0)
Abdomen	15.8 (10.8–21.3)
Thigh	8.7 (7.0–10.8)
Calf	5.1 (4.3–6.5)

**Table 4:** Body fat percentage measured with DXA and estimated with anthropometric equations developed in athletic populations.

Author equation	Median (25th–75th percentile)
DXA	14.0 (11.9–16.4)
Thorland (3SKF)	9.2 (6.3–12.0)^*∗∗*^
White	9.4 (7.4–11.3)^*∗∗*^
Stewart	10.2 (7.2–13.3)^*∗∗*^
Withers	10.5 (8.6–13.1)^*∗∗*^
Evans (3SKF)	10.5 (8.9–12.7)^*∗∗*^
Pascale	10.5 (9.3–12.2)^*∗∗*^
Reilly	10.7 (9.6–12.2)^*∗∗*^
Thorland (7SKF)	10.8 (7.8–14.4)^*∗∗*^
Evans (7SKF)	11.2 (9.4–13.5)^*∗∗*^
Civar	12.6 (10.5–14.5)
Forsyth (4SKF)	13.0 (9.6–17.9)
Oliver	13.1 (11.0–15.5)
Forsyth (2SKF)	13.7 (10.1–18.4)
Zuti	14.3 (12.5–17.6)

2SKF, two skinfolds; 3SKF, three skinfolds; 4SKF, four skinfolds; 7SKF, seven skinfolds; DXA, dual-energy X-ray absorptiometry. Different compared with DXA.  ^*∗∗*^*p* < 0.001.

**Table 5:** Body fat percentage differences (equation − DXA) from anthropometric equations (developed in athletic populations) statistically similar to DXA.

Author equation	Mean	95% limits of agreement	*r* ^*∗*^
Oliver	−0.8	(−3.9 to 2.3)	−0.29
Civar	−1.5	(−4.8 to 1.8)	−0.55
Zuti	0.7	(−4.8 to 6.3)	−0.31
Forsyth (4SKF)	−0.3	(−6.2 to 5.7)	0.45
Forsyth (2SKF)	0.3	(−5.8 to 6.5)	0.44

Equations are listed from the narrowest to the widest limits of agreement. 2SKF, two skinfolds; 4SKF, four skinfolds; DXA, dual-energy X-ray absorptiometry. ^*∗*^All *r* values were significant (*p* < 0.001).

**Table 6:** Body fat percentage measured with DXA and estimated with anthropometric equations developed in nonathletic populations.

Author equation	Median (25th–75th percentile)
DXA	14.0 (11.9–16.4)
Lohman	8.0 (7.1–8.8)^*∗∗*^
Sloan	8.4 (6.8–10.1)^*∗∗*^
Pollock	8.4 (7.1–10.0)^*∗∗*^
Jackson (3SKF)	8.9 (6.7–12.1)^*∗∗*^
Jackson (7SKF)	9.8 (7.3–12.6)^*∗∗*^
Katch	10.4 (8.7–12.9)^*∗∗*^
Eston	11.0 (9.3–13.0)^*∗∗*^
Nagamine	12.0 (10.4–13.6)^*∗*^
Wilmore	13.4 (11.4–15.8)
Ball	13.7 (11.5–16.4)
Lean	14.5 (11.1–17.3)
Garcia	14.9 (11.7–18.2)
Van der Ploeg	15.1 (12.5–18.1)
Durnin-W	15.3 (12.2–18.1)
Durnin-R	15.8 (12.7–18.1)
Leahy	16.4 (13.7–19.9)^*∗*^
Peterson	17.9 (15.1–20.5)^*∗∗*^

3SKF, three skinfolds; 7SKF, seven skinfolds; DXA, dual-energy X-ray absorptiometry. Different compared with DXA.   ^*∗*^*p* < 0.02; ^*∗∗*^*p* < 0.001.

**Table 7:** Body fat percentage differences (equation − DXA) from anthropometric equations (developed in nonathletic populations) statistically similar to DXA.

Author equation	Mean	95% limits of agreement	*r*
Ball	−0.2	(−3.4 to 3.1)	−0.15
Wilmore	−0.4	(−3.9 to 3.0)	−0.51^*∗*^
Durnin-R	1.4	(−2.3 to 5.1)	−0.11
Van der Ploeg	1.3	(−2.4 to 5.1)	0.12
Durnin-W	1.1	(−3.0 to 5.2)	−0.09
Lean	0.2	(−4.5 to 5.0)	0.11
Garcia	0.3	(−4.7 to 5.2)	0.09

Equations are listed from the narrowest to the widest limits of agreement. DXA, dual-energy X-ray absorptiometry. ^*∗*^Significant correlation (*p* < 0.001).
